# Dr. George F. Harris

**Published:** 1918-05

**Authors:** 


**Dr. George F. Harris,** Buffalo; Cornell University Medical College, New York City, 1904. He was a member of the medical staff of the Buffalo State Hospital. He died March 18.

